# A Standardized Approach to Reduce Fluid Overload in Critically Ill Children

**DOI:** 10.1097/pq9.0000000000000813

**Published:** 2025-05-01

**Authors:** Andrew J. Hopwood, Tina M. Schade Willis, Michelle C. Starr, Katie M. Hughes, Stefan W. Malin

**Affiliations:** From the * Division of Critical Care, Department of Pediatrics, Indiana University School of Medicine, Indianapolis, Ind.; † Division of Pediatric Nephrology, Department of Pediatrics, Indiana University School of Medicine, Indianapolis, Ind.

## Abstract

**Introduction:**

Fluid overload, the pathologic state of positive fluid balance, is common in the pediatric intensive care unit (PICU) and is independently associated with poor outcomes. Quality improvement–based processes to measure and assess fluid balance in critically ill children are lacking.

**Methods:**

The primary aim was to develop and implement a fluid management strategy that includes the standardized measurement and assessment of fluid balance, which is adhered to in at least 50% of all PICU patients. The 4 components of the strategy include (1) creating a fluid balance dashboard that tracks percent cumulative fluid balance over time, (2) documentation of daily weights, (3) fluid balance reporting and discussion incorporated into standardized rounds, and (4) active total intravenous (IV) fluid order.

**Results:**

We reviewed 280 patient encounters between May 2023 and April 2024 and achieved the primary aim of at least 50% compliance with the fluid management strategy and maintained this success over time. Achieving the primary aim coincides with implementing daily weights and total IV fluid orders into PICU admission order sets.

**Conclusions:**

In this quality improvement project, we develop, implement, and maintain compliance with a fluid management strategy. Future work will involve daily utilization of the fluid balance dashboard and monitoring compliance with total IV fluid orders. Implementing a quality improvement–based fluid management strategy may lead to improved awareness of the fluid status of patients and the prescription of fluid therapy to mitigate the harmful effects of fluid overload.

## INTRODUCTION

Fluid overload, the pathologic state of positive fluid balance, is common in the pediatric intensive care unit (PICU) and is independently associated with adverse outcomes, including prolonged mechanical ventilation, acute kidney injury, and mortality.^1–11^ Multiple guidelines, including those for pediatric acute respiratory distress syndrome and those treated with extracorporeal membrane oxygenation, emphasize the importance of fluid balance monitoring.^12,13^

Many pediatric critical care physicians prescribe maintenance fluids using the Holliday-Segar (“4-2-1”) method.^14^ However, critically ill children often receive continuous intravenous (IV) fluids above maintenance requirements, a phenomenon referred to as “fluid creep.”^15–20^ One study of children admitted to the PICU reported that a higher percent cumulative fluid balance correlated with higher odds of mortality and identified nonresuscitation fluid above hydration requirements as a potentially modifiable risk factor.^16^

The absence of standardized processes in measuring and assessing fluid balance and administering IV fluids in critically ill children underscores the need for standardization utilizing quality improvement methodology.^21^ This quality improvement project aimed to develop and implement a fluid management strategy, including the standardized measurement and assessment of fluid balance and administration. The goal is compliance with the strategy in at least 50% of patients in the PICU at our institution within 12 months.

## METHODS

### Context

We implement this single-center quality improvement project in the PICU, excluding the separate cardiovascular unit, at a free-standing quaternary referral children’s hospital affiliated with a large academic medical center. The hospital is a level 1 trauma center and Extracorporeal Life Support Organization Platinum Center of Excellence. The 36-bed PICU cares for a wide range of surgical and medical patients, averaging 2,500 admissions annually. The hospital utilizes Cerner (Oracle Corp., Redwood Shores, Calif.) as its electronic health record (EHR).

### Determination of Cumulative Fluid Balance

Two commonly used approaches to assess a patient’s cumulative fluid balance are the fluid intake-output and weight-based methods.^21^ Calculations for each method are as follows:

Fluid intake-output method: $\%CFB\left(fluid\right)=\left[\frac{Fluid\;intake\left(L\right)-Fluid\;output\left(L\right)}{PICU\;admission\;weight\left(kg\right)}\right]\times 100$_._

Weight-based method: %CFB(weight)=$\left[\frac{Most\;recent\;weight\left(kg\right)-PICU\;admission\;weight\left(kg\right)}{PICU\;admission\;weight\left(kg\right)}\right]$×100_._

The weight-based method may better account for insensible losses and compensate for inaccurate intake and output data utilized in the fluid intake-output method. The fluid intake-output method is usually more practical for calculation, as the data are readily available in the PICU. In contrast, weight data are not always readily available and are subject to measurement error.

### Intervention

**Fig. 1.**
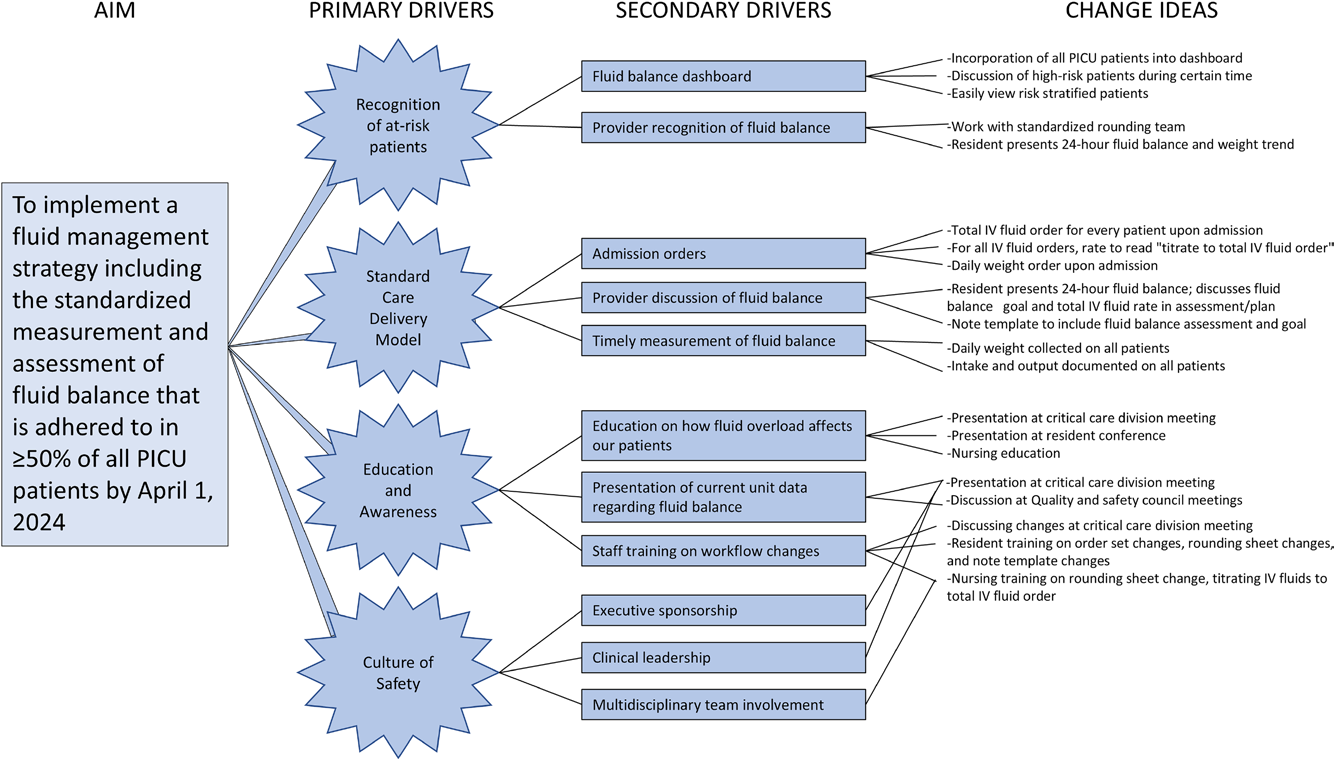
Key driver diagram displaying the primary drivers, secondary drivers, and change ideas that contribute to achieving the primary aim.

The primary aim was to develop and implement a fluid management strategy to standardize the measurement and assessment of fluid balance and administration in critically ill children. A multidisciplinary team, including a pediatric critical care fellow, pediatric intensivists, a pediatric nephrologist, a PICU pharmacist, and PICU nurses, met to develop the strategy. A key driver diagram (Fig. 1) was utilized to prioritize efforts and convey the team’s shared view to stakeholders in the PICU. The strategy has four components; a patient must meet all 4 to be compliant.

#### Component 1: Presence of Fluid Balance Dashboard That Tracks Percent Cumulative Fluid Balance over Time

**Fig. 2.**
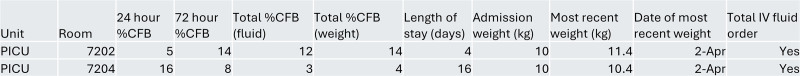
Example showing 2 patients on the fluid balance dashboard. “24-hour %CFB” and “72-hour %CFB” utilize the fluid intake-output method to calculate the percent cumulative fluid balance over the past 24 and 72 hours, respectively. “Total %CFB (fluid)” represents the patient’s percent cumulative fluid balance utilizing the fluid intake-output method over the entire hospital stay. In contrast, “Total %CFB (weight)” utilizes the weight-based method to calculate the percent cumulative fluid balance during the entire hospital stay. “Admission weight” and “Most recent weight” columns help add context. The “Date of most recent weight” column and “Total IV fluid order” columns help ease rounding audits, allowing the project team to easily visualize whether a patient has had a weight in the past 24 hours and an active total fluid order. CFB, cumulative fluid balance.

A fluid balance dashboard was created using Microsoft Power BI (Microsoft Corp., Redmond, Wash.). The tool organizes data from the EHR to create a dashboard utilizing real-time data. The dashboard provides clinically actionable information on the patient’s percent cumulative fluid balance over various time points and with different methods. Figure 2 provides an example of the dashboard.

#### Component 2: Documented Daily Weight

Obtaining consistent daily weights is a unit priority tracked by the PICU quality and safety team (consisting of the PICU quality and safety physician leader, PICU nurse leaders, PICU medical director, and PICU pharmacists). Our unit follows a standard process for obtaining daily weights (with the patient’s daily bath), and recurring education for bedside nursing focuses on the importance of obtaining daily weights. We also standardize PICU admission order sets to include an order to obtain a weight every 24 hours rather than the previous order for twice weekly weights.

#### Component 3: Fluid Balance Reporting and Discussion Incorporated into Standardized Rounds

The daily bedside rounding sheet utilized by trainees presenting patients in the PICU now includes a more detailed discussion of fluid balance: 24-hour intake and output, daily weight, the total IV fluid rate, and a daily fluid balance goal. PICU trainee orientation now includes education regarding our standardized rounding process, including fluid balance.

#### Component 4: Active Total IV Fluid Order If Receiving Continuous IV Fluids

PICU admission order sets now include a “total IV fluid order.” This order specifies a total rate at which all continuous fluids should run, including language on how to titrate fluids as patients start enteral feeds. We provide education on the total IV fluid order to physicians and nurses. This order was first trialed in patients with *status asthmaticus*, as they have a specific PICU admission order set. Plan-do-study-act cycles during the trial included updating our commonly used IV fluid orders so that the fluid rate reads to titrate to total IV fluid order, adjusting language in the total IV fluid order for clarity, and expanding bedside staff education before modifying all PICU admission order sets to contain a total IV fluid order. We did not dictate fluid therapy; providers could use any fluid composition or infusion rate.

### Evaluation of the Intervention

We chose a strategy similar to evidence-based care bundles^22^ to evaluate compliance with the fluid management strategy. Compliance is important because some patients may meet 1 or 2 components; however, compliance is not uniform or consistent. The goal was to combine the interventions into a fluid management strategy used consistently for all patients. This approach makes assessing compliance with the fluid management strategy a straightforward “yes” or “no.”

### Measures and Analysis

The primary aim was compliance with the fluid management strategy in greater than or equal to 50% of all PICU patients within one year. Each patient encounter must meet all four components to comply with the strategy. The operational definitions for the components (or process measures) previously described are as follows:

1. The patient is active on the fluid balance dashboard, obtained by viewing the fluid balance dashboard.
2. The patient has a weight documented within the past 24 hours, obtained by viewing the fluid balance dashboard.
3. The patient’s fluid balance is discussed on rounds. For compliance, a member of the rounding team must report the past 24-hour fluid balance or change in weight during daily PICU rounds.
4. The patient has an active total IV fluid order if receiving continuous (commonly referred to as maintenance) IV fluids, obtained by viewing the fluid balance dashboard.

One project team member obtained data regarding each component in real-time during PICU rounds and recorded it on a standardized reporting form.

Balancing measures for this project include accidental endotracheal tube dislodgement while obtaining a patient weight and duration of PICU rounds. Some staff expressed concern about endotracheal tube dislodgement while weighing a patient, especially if weighing all patients daily. The duration of PICU rounds may be increased if we achieve our goal of increasing the frequency of fluid balance reporting. Our interventions are occurring concurrently with changes being made to standardized rounds at our institution, so it will be difficult to say if a change in the duration of rounds is due to our interventions. Information regarding unplanned extubations and the duration of rounds has already been collected at our institution.

Statistical process control (SPC) p charts were used to evaluate the primary outcome and process measures over time.^23^ Data were collected from May 2023 to April 2024 and reported in groups of 10 patients. All SPC charts were created using QI Macros (KnowWare International, Denver, Colo.). We follow Standard for Quality Improvement Reporting Excellence guidelines for reporting this project.^24^

The Indiana University institutional review board determined this project was not human subjects research (IRB no. 18988).

### Secondary Outcomes

Secondary outcome data were obtained in all mechanically ventilated patients without a tracheostomy in the PICU from January 2023 to May 2024. We chose this period to evaluate outcomes before and after compliance with the fluid management strategy. We chose mechanically ventilated patients as they were most at risk for fluid overload and likely to benefit from our approach. When neonates at our institution undergo tracheostomy, they are transferred to the PICU. Given the significant number of these patients and the length of hospital stay and duration of mechanical ventilation in this population, their inclusion likely would have skewed our secondary outcomes. We obtain the duration of mechanical ventilation, PICU length of stay, mortality, and pediatric risk of mortality (PRISM) score from the Virtual Pediatric Systems database. We obtain the percentage of cumulative fluid balance at continuous renal replacement therapy (CRRT) initiation from an internal database. Run charts created with QI Macros (KnowWare International) display the duration of mechanical ventilation and length of stay.

## RESULTS

**Fig. 3.**
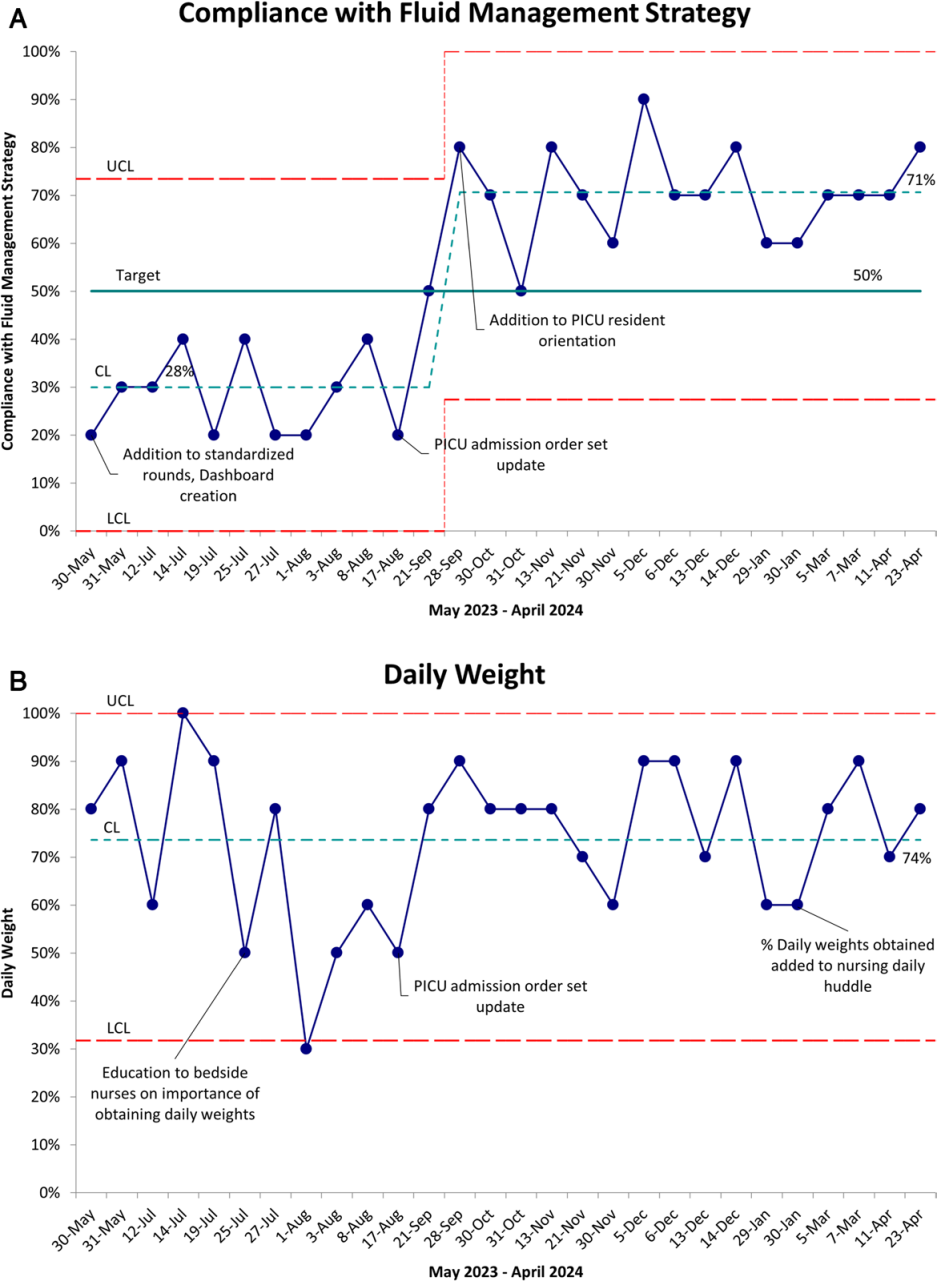
Primary outcome and process measure SPC charts. A, SPC p chart showing percentage compliance with fluid management strategy over time. Since September 2023, the CL has shifted up from 28% to 71%, above the target of 50%. B, SPC p chart showing the percentage of patients with a daily weight, with a CL around 74%. CL, center line; LCL, lower control limit; UCL, upper control limit.

**Fig. 4.**
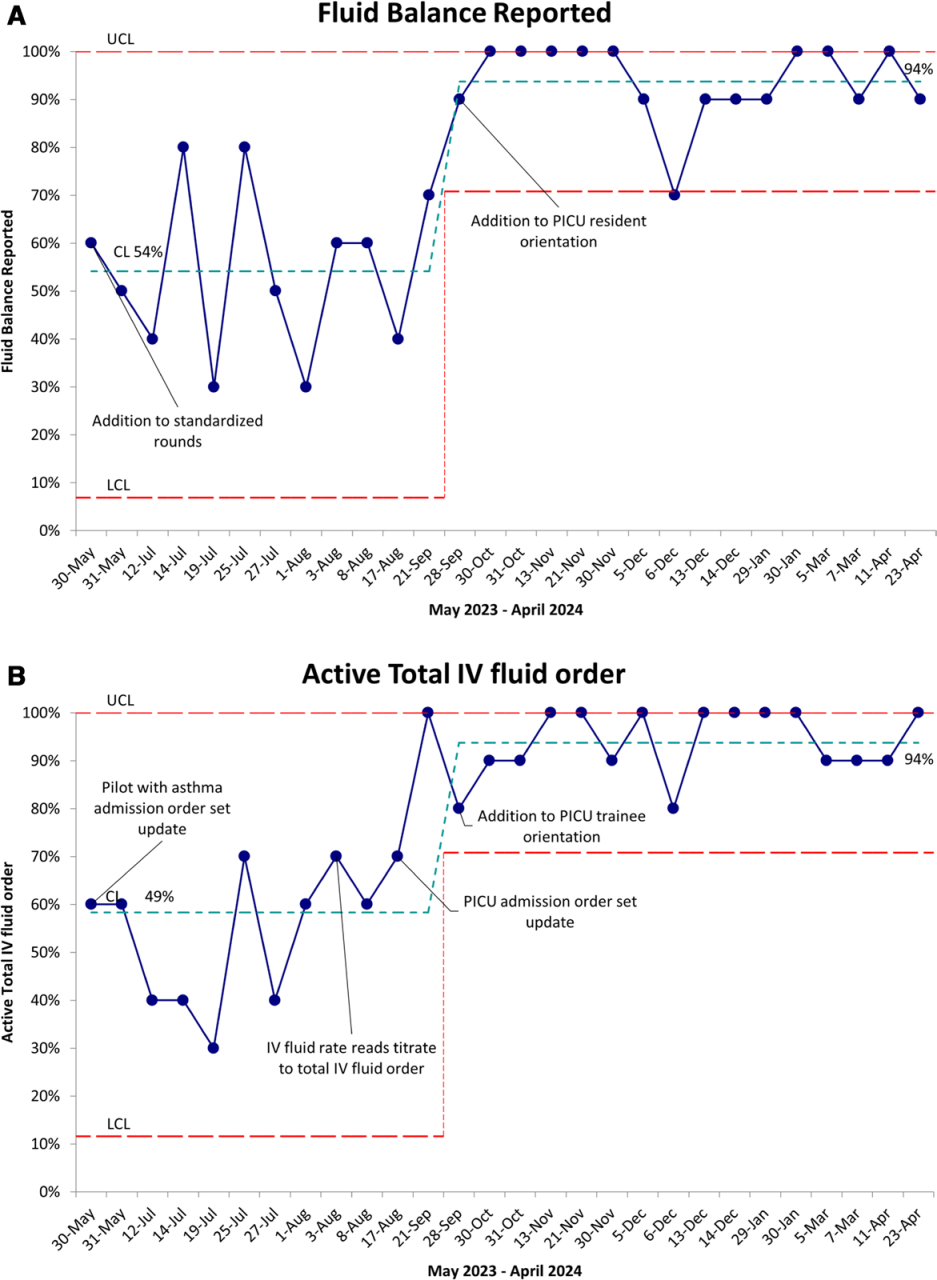
Primary outcome and process measure SPC charts. A, SPC p chart showing the percentage of patients with fluid balance discussed on rounds. Since September 2023, the CL has shifted from 54% to 94%. B, Run chart showing the percentage of patients with an active IV fluid order while receiving continuous IV fluids. Since September 2023, the CL has increased from 49% to 94%. CL, center line; LCL, lower control limit; UCL, upper control limit.

A total of 280 patient encounters were reviewed between May 2023 and April 2024. Since September 2023, we have achieved the primary aim of greater than or equal to 50% compliance with the fluid management strategy and maintained this success over time (Fig. 3A). This 50% or greater compliance timing coincided with including daily weights and total IV fluid orders into PICU admission order sets. Component 2 (documentation of a daily weight) showed a wide variance throughout the study period, with a center line of 74% (Fig. 3B). Since updating PICU resident orientation, component 3 (the report of fluid balance on daily PICU rounds) has shown a shift from 54% to 94% (Fig. 4A). After implementing the updated admission order set, the center line for component 4 (patients with total IV fluid orders receiving continuous IV fluids) shifted from 49% to 94% (Fig. 4B). Regarding balancing measures, upon review of the unplanned extubation reports, none of the unplanned extubations in the PICU from April 2023 to May 2024 were attributed to movement while weighing a patient. Data regarding the duration of rounds were collected from April 2023 to November 2023; over that time, the duration of rounds displayed little variation.

**Fig. 5.**
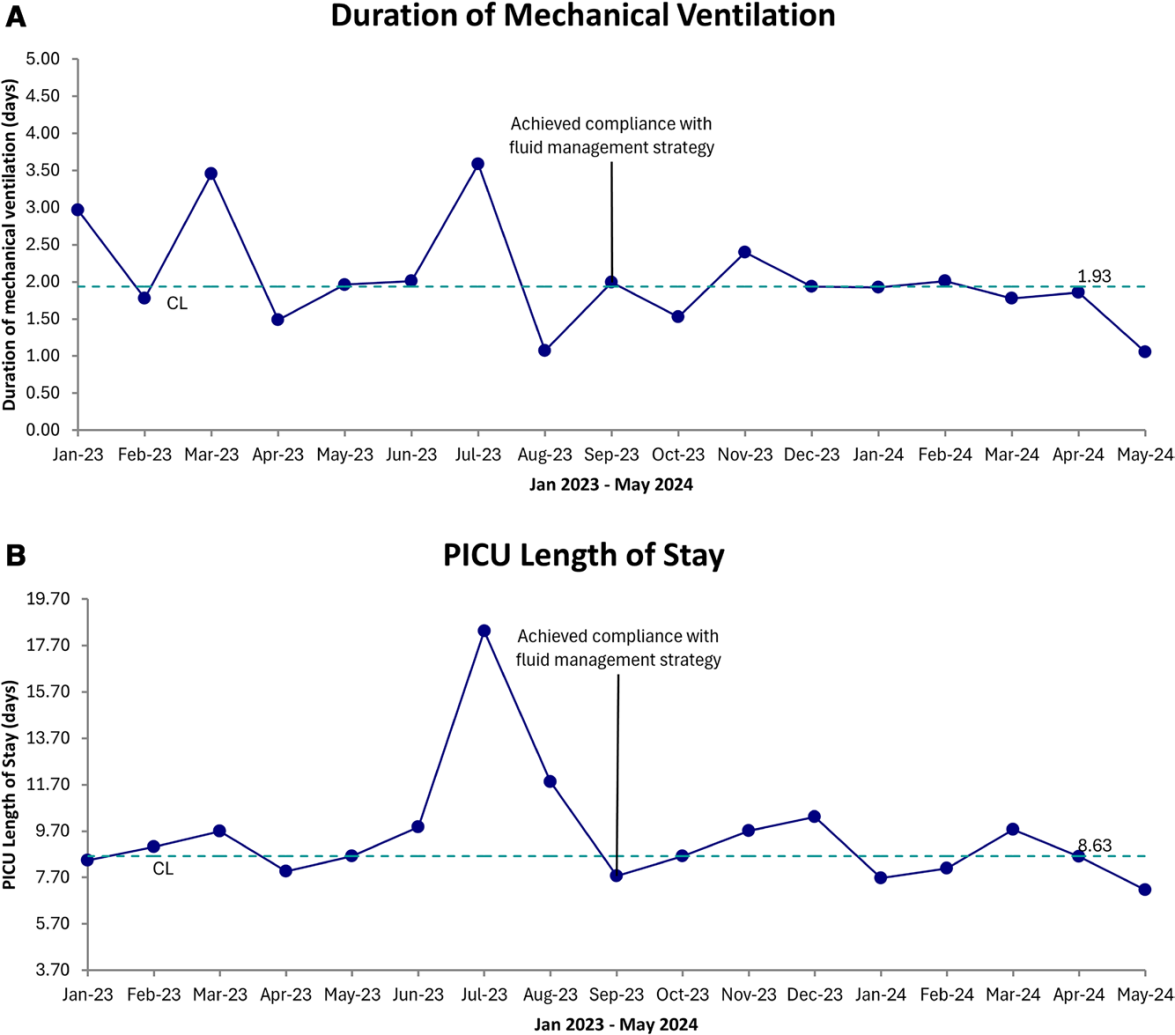
Secondary outcome run charts. A, Run chart displaying the duration of mechanical ventilation by month. B, Run chart displaying PICU length of stay by month. CL, center line.

We evaluated secondary outcomes before and after compliance with the fluid management strategy in 790 patients. There were 371 patients in the before group and 419 patients in the after group. Median values for all secondary outcomes are displayed (Table 1). The duration of mechanical ventilation, PICU length of stay, and percent cumulative fluid balance at CRRT initiation decreased in the after group. Mortality was similar between the before and after groups. The PRISM score, a measure of illness severity, was similar between the before and after groups. We display the duration of mechanical ventilation (Fig. 5A) and PICU length of stay (Fig. 5B) over the prespecified period with run charts, during which there were no significant shifts.

**Table 1.** Secondary Outcomes before and after Compliance with Fluid Management Strategy

	Before Compliance	After Compliance
Duration of mechanical ventilation, d (IQR)	2.08 (0.86–5.71)	1.82 (0.72–4.86)
PICU length of stay, d (IQR)	5.07 (2.35–11.04)	3.88 (1.74–10.76)
Percent cumulative fluid balance at CRRT initiation, %	10.8	8.9
Mortality, % (IQR)	9.94 (6.62–11.75)	9.76 (8.23–13.53)
PRISM score (IQR)	6.78 (0–9)	6.22 (0–8)

Values expressed as medians.

IQR, interquartile range.

## DISCUSSION

This ongoing single-center quality improvement study achieves compliance with a fluid management strategy in the PICU. Standardizing the approach to measuring and assessing fluid balance and administration decreases unnecessary variation in fluid balance reporting and utilization of total IV fluid orders. This approach will increase the availability of data needed to make clinical decisions on fluid management and improve the recognition of patients developing a pathologically positive fluid balance, potentially allowing care providers to intervene before complications of fluid overload develop.

The intervention that led to the largest improvement was modifying PICU admission order sets to include an order for daily weights and the total IV fluid order. These changes resulted in sustained improvement throughout the study period. Codifying changes in workflow, such as updating admission order sets, was crucial to our success.

Component 2 (documentation of daily weights) shows the greatest variation among the components. Another study implementing daily weight orders demonstrates variation in the frequency of obtaining weights, though with less overall adherence.^25^ Partnering with physician and nursing leadership teams is imperative to sustain improvement. Education results in a shared understanding of how daily weights inform clinical practice. Bedside nurses report that the discussion of weights in daily rounds and utilization of weight data in clinical decision-making are the most impactful in helping them remember to obtain patients’ daily weights. In patients with missing daily weights, the most reported reason is a lack of clarity regarding when to obtain a weight. Work to reinforce when to obtain weights (with the patient’s daily bath) further standardizes the process of obtaining daily weights, which may vary between institutions. After September 2023, a missing daily weight was the most common reason a patient would be labeled noncompliant with the strategy. Future work will focus on standardizing the scales used for each patient and how to obtain weight measurements.^26^ A reliable weight is one measured using the same method and scale, as precise measurements are essential for assessing a patient’s fluid status.^21^

Component 3 (discussion of fluid balance during rounds) improved and sustained around 94%. In preparation for this intervention, our center conducted a study evaluating how often we discuss fluid balance and recognize fluid overload. For patients in the PICU, fluid status was explicitly discussed in only 54%. In children with at least 10% positive cumulative fluid balance, clinicians recognized fluid overload in only 26%.^27^ How often providers discuss fluid balance and recognize fluid overload may depend upon the rounding structure and presenter. For example, in one tertiary academic medical ICU where physician trainees are the primary presenters, 67% of trainees omitted data regarding fluid balance.^28^ To improve the discussion of fluid balance on rounds, we worked with our PICU standardized rounds group to include the 24-hour fluid balance, total IV fluid rate, and fluid balance goal to be presented on rounds daily. We observed the most drastic improvement after updating PICU resident orientation to include fluid balance as a component of standardized rounds and education regarding its importance. Future work will involve increasing how often the team reports the total IV fluid rate and fluid balance goal for the patient on rounds.

Component 4 (the presence of a total IV fluid order for patients receiving continuous IV fluids) sustained around 94% after the change in admission orders. We piloted this change along with the order for daily weights in *status asthmaticus* admissions. Changes to our institutional EHR are best to trial in a smaller subset to ensure no technical issues with the update. This pilot allowed us to identify the need to update other IV fluid orders to limit the creation of extra work on the ordering provider. An order set already existed for the most used IV fluids in the PICU, so we changed the default fluid rate to “titrate to total IV fluid order.” Now, the ordering provider types in the fluid rate they desired in the total IV fluid order, allowing nursing to titrate the continuous maintenance fluid to the desired rate. We also updated the language regarding the initiation of enteral nutrition, so there was no limitation on enteral feeds due to misunderstandings regarding the total IV fluid order. After instituting the total IV fluid order, observations demonstrated that it was not always correctly followed. Therefore, future work will focus on improving adherence to the total IV fluid order.

The fluid balance dashboard remains the most innovative element of the fluid management strategy, as it allows tracking process measures in real time. Future work will involve the operationalization of the dashboard in clinical practice. This effort will provide valuable insights into fluid status by making the percent cumulative fluid balance easily available to providers, including at clinically relevant time points such as the past 24 and 72 hours. The dashboard can potentially assist in clinical decision-making algorithms to develop interventions based on fluid balance. For example, providers can easily identify thresholds of percent cumulative fluid balance (5%–10%, 10%–20%, and >20%), making them aware of this important clinical information. Having the dashboard in place will assist with future research, such as active fluid removal strategies at certain percent cumulative fluid balance thresholds. Given the flexible nature of the dashboard, it will be easy to incorporate future variables as the field discovers them to be clinically relevant.

We evaluate secondary outcomes before and after compliance with the fluid management strategy. When comparing the before group to the after group, there was a decrease in the duration of mechanical ventilation, PICU length of stay, and percent cumulative fluid balance at CRRT initiation. The target of this standardized fluid balance measurement and assessment approach is not associated with improvements in secondary outcomes. Instead, this serves as a foundation of standardized processes that have not previously existed, allowing for the appropriate investigations to improve outcomes.

### Limitations

Despite the strengths of this study, it has several limitations. Although most PICU physicians agreed to an active total IV fluid order, we did not mandate a specific rate for all patients. Next, interventions focusing on daily weights depend on institutional commitment, as obtaining weights requires leadership buy-in at multiple levels if daily weights are not common unit practice. Finally, given collecting data for fluid balance reporting required the researcher’s physical presence on rounds, the teams could not be blinded, leading to a potential Hawthorne effect. Therefore, one cannot say if fluid balance reporting on rounds is as robust when not directly observed.

## CONCLUSIONS

The primary aim of at least 50% compliance with our fluid management strategy has been achieved and sustained since September 2023. There is potential for expansion to the cardiovascular ICU at our institution, adult ICUs within our health system, and multicenter involvement with PICUs nationally and internationally. This work will help improve the care of critically ill children by standardizing the measurement and assessment of fluid balance and administering fluids, which should improve provider awareness of fluid balance and combat fluid creep. This strategy will also lay the groundwork for future research regarding fluid balance and fluid overload in children by creating a system to monitor the fluid balance of our patients and contribute to data needed to help develop clinical decision tools for managing fluid balance at the bedside.

## References

[1] Alobaidi R Basu RK DeCaen A. Fluid accumulation in critically ill children. Crit Care Med. 2020;48:1034–1041. 32371612 10.1097/CCM.0000000000004376

[2] Alobaidi R Morgan C Basu RK. Association between fluid balance and outcomes in critically ill children: a systematic review and meta-analysis. JAMA Pediatr. 2018;172:257–268. 29356810 10.1001/jamapediatrics.2017.4540 PMC5885847

[3] Arikan AA Zappitelli M Goldstein SL. Fluid overload is associated with impaired oxygenation and morbidity in critically ill children. Pediatr Crit Care Med. 2012;13:253–258. 21760565 10.1097/PCC.0b013e31822882a3

[4] Diaz F Benfield M Brown L. Fluid overload and outcomes in critically ill children: a single center prospective cohort study. J Crit Care. 2017;39:209–213. 28254390 10.1016/j.jcrc.2017.02.023

[5] Flores-Gonzalez JC Valladares CM Yun Castilla C; Bronquiolitis en la Unidad de Cuidados Intensivos Pediátricos (BRUCIP) WorkGroup. Association of fluid overload with clinical outcomes in critically ill children with bronchiolitis: bronquiolitis en la Unidad de Cuidados Intensivos Pediatricos (BRUCIP) Study. Pediatr Crit Care Med. 2019;20:e130–e136. 30664037 10.1097/PCC.0000000000001841

[6] Gelbart B Serpa Neto A Stephens D. Fluid accumulation in mechanically ventilated, critically ill children: retrospective cohort study of prevalence and outcome. Pediatr Crit Care Med. 2022;23:990–998. 36454001 10.1097/PCC.0000000000003047

[7] Mah KE Hao S Sutherland SM. Fluid overload independent of acute kidney injury predicts poor outcomes in neonates following congenital heart surgery. Pediatr Nephrol. 2018;33:511–520. 29128923 10.1007/s00467-017-3818-x

[8] Selewski DT Askenazi DJ Bridges BC. The impact of fluid overload on outcomes in children treated with extracorporeal membrane oxygenation: a multicenter retrospective cohort study. Pediatr Crit Care Med. 2017;18:1126–1135. 28937504 10.1097/PCC.0000000000001349 PMC5716912

[9] Valentine SL Sapru A Higgerson RA; Pediatric Acute Lung Injury and Sepsis Investigator's (PALISI) Network. Fluid balance in critically ill children with acute lung injury. Crit Care Med. 2012;40:2883–2889. 22824936 10.1097/CCM.0b013e31825bc54d PMC3455114

[10] Matsushita FY Krebs VLJ de Carvalho WB. Association between fluid overload and mortality in newborns: a systematic review and meta-analysis. Pediatr Nephrol. 2022;37:983–992. 34727245 10.1007/s00467-021-05281-8

[11] Messmer AS Zingg C Muller M. Fluid overload and mortality in adult critical care patients-a systematic review and meta-analysis of observational studies. Crit Care Med. 2020;48:1862–1870. 33009098 10.1097/CCM.0000000000004617

[12] Emeriaud G Lopez-Fernandez YM Iyer NP; Second Pediatric Acute Lung Injury Consensus Conference (PALICC-2) Group on behalf of the Pediatric Acute Lung Injury and Sepsis Investigators (PALISI) Network. Executive summary of the second international guidelines for the diagnosis and management of Pediatric Acute Respiratory Distress Syndrome (PALICC-2). Pediatr Crit Care Med. 2023;24:143–168. 36661420 10.1097/PCC.0000000000003147 PMC9848214

[13] Bridges BC Dhar A Ramanathan K. Extracorporeal life support organization guidelines for fluid overload, acute kidney injury, and electrolyte management. ASAIO J. 2022;68:611–618. 35348527 10.1097/MAT.0000000000001702

[14] Hassinger AB Valentine SL. Self-Reported management of IV fluids and fluid accumulation in children with acute respiratory failure. Pediatr Crit Care Med. 2018;19:e551–e554. 30074980 10.1097/PCC.0000000000001685

[15] Al-Lawati ZH Sur M Kennedy CE. Profile of fluid exposure and recognition of fluid overload in critically ill children. Pediatr Crit Care Med. 2020;21:760–766. 32168295 10.1097/PCC.0000000000002337

[16] Barhight MF Nelson D Chong G. Non-resuscitation fluid in excess of hydration requirements is associated with higher mortality in critically ill children. Pediatr Res. 2022;91:235–240. 33731814 10.1038/s41390-021-01456-z PMC7968408

[17] Orwoll KRB. Fluid creep in the PICU: characterizing fluid administration beyond maintenance fluids. Pediatrics. 2021;147:464–465.

[18] Fuhrman D Crowley K Vetterly C. Medication use as a contributor to fluid overload in the PICU: a prospective observational study. J Pediatr Intensive Care. 2018;7:69–74. 31073473 10.1055/s-0037-1604422 PMC6260341

[19] Gamble KC Smith SE Bland CM. Hidden fluids in plain sight: identifying intravenous medication classes as contributors to intensive care unit fluid intake. Hosp Pharm. 2022;57:230–236. 35601708 10.1177/00185787211016339 PMC9117780

[20] Langer T D’Oria V Spolidoro GCI. Fluid therapy in mechanically ventilated critically ill children: the sodium, chloride and water burden of fluid creep. BMC Pediatr. 2020;20:424. 32891127 10.1186/s12887-020-02322-3 PMC7487923

[21] Selewski DT Barhight MF Bjornstad EC; Pediatric the Acute Disease Quality Initiative (ADQI) Consensus Committee Members. Fluid assessment, fluid balance, and fluid overload in sick children: a report from the Pediatric Acute Disease Quality Initiative (ADQI) conference. Pediatr Nephrol. 2024;39:955–979. 37934274 10.1007/s00467-023-06156-w PMC10817849

[22] Horner D Bellamy M. Care bundles in intensive care. BJA Educ. 2012;12:199–202.

[23] Provost LP Murray SK. The Health Care Data Guide: Learning from Data for Improvement, 2nd ed. Jossey-Bass; 2022.

[24] Ogrinc G Davies L Goodman D. SQUIRE 2.0 (Standards for QUality Improvement Reporting Excellence): revised publication guidelines from a detailed consensus process. BMJ Qual Saf. 2016;25:986–992. 10.1136/bmjqs-2015-004411 PMC5256233 26369893

[25] Ahearn MA Soranno DE Stidham T. Adherence to daily weights and total fluid orders in the pediatric intensive care unit. Pediatr Qual Saf. 2018;3:e110. 30584637 10.1097/pq9.0000000000000110 PMC6221598

[26] Srinivasan V Seiple S Nagle M. Improving the performance of anthropometry measurements in the pediatric intensive care unit. Pediatr Qual Saf. 2017;2:e022. 30229160 10.1097/pq9.0000000000000022 PMC6132458

[27] Starr MC Weston E Chmielewski J. Identification of fluid overload in critically ill children: fluid status, the missing vital sign. J Transl Crit Care Med. 2024;6:e23–00009.

[28] Artis KA Bordley J Mohan V. Data omission by physician trainees on ICU rounds. Crit Care Med. 2019;47:403–409. 30585789 10.1097/CCM.0000000000003557 PMC6407821

